# Evolutionary Conservation and Network Structure Characterize Genes of Phenotypic Relevance for Mitosis in Human

**DOI:** 10.1371/journal.pone.0036488

**Published:** 2012-05-02

**Authors:** Marek Ostaszewski, Serge Eifes, Antonio del Sol

**Affiliations:** Luxembourg Centre for Systems Biomedicine, Luxembourg, Luxembourg; Centro de Investigación Príncipe Felipe, Spain

## Abstract

The impact of gene silencing on cellular phenotypes is difficult to establish due to the complexity of interactions in the associated biological processes and pathways. A recent genome-wide RNA knock-down study both identified and phenotypically characterized a set of important genes for the cell cycle in HeLa cells. Here, we combine a molecular interaction network analysis, based on physical and functional protein interactions, in conjunction with evolutionary information, to elucidate the common biological and topological properties of these key genes. Our results show that these genes tend to be conserved with their corresponding protein interactions across several species and are key constituents of the evolutionary conserved molecular interaction network. Moreover, a group of bistable network motifs is found to be conserved within this network, which are likely to influence the network stability and therefore the robustness of cellular functioning. They form a cluster, which displays functional homogeneity and is significantly enriched in genes phenotypically relevant for mitosis. Additional results reveal a relationship between specific cellular processes and the phenotypic outcomes induced by gene silencing. This study introduces new ideas regarding the relationship between genotype and phenotype in the context of the cell cycle. We show that the analysis of molecular interaction networks can result in the identification of genes relevant to cellular processes, which is a promising avenue for future research.

## Introduction

In the field of of systems biology, understanding the relationship between genotype and phenotype within the context of biological networks represents a major goal. Over the next few years, the number of completely sequenced individual human genomes will grow tremendously, and a key challenge will be to understand the link between genotypic and phenotypic variation in health and disease states [1].

One of the possible experimental approaches, which may be used to establish a phenotype-genotype relationship, is gene perturbation screens by silencing or overexpression, coupled with the observation of the phenotypic effects. An important goal is to find associations between the different gene perturbation screens to identify molecular interactions, cellular pathways and regulatory mechanisms [2]. System-level approaches (particularly, network analyses) are appropriate methods to address this issue.

Moreover, an evolutionary cross-species comparison may provide a framework to address these challenges and allow researchers to draw generalized conclusions about genotype-phenotype relationships. Different studies have highlighted the importance of characterizing genotype-phenotype relationships in an evolutionary context [3]–[6]. Even though evolutionary conservation may be important for delineating the phenotypic properties in living organisms, there is no simple and consistent relationship between homologous genes and the morphological structures, in which they are implicated [7].

Evolutionary conservation can be considered at different levels of molecular interaction networks (e.g. genes, protein interactions and network motifs). At the genetic level this conservation suggests retention of functional aspects. At the protein interaction level, several studies have focused on understanding the evolutionary and structural aspects of network topology [8]–[11] and have indicated co-conservation of physically interacting proteins [10], [12], [13]. Network motifs represent functional entities of cellular networks that are evolutionary conserved [14]. Conserved network motifs were used for the prediction of protein interactions [15]. In a similar context, cross-species comparison of global protein interactomes revealed conserved subnetworks, which are useful for predicting protein interactions and functions [16], [17].

Here, we carried out evolutionary and network analyses to characterize the genes involved in phenotypic aberrations of mitosis in human. Our study was based on a recent genome-wide experimental study by Neumann et al. [18], which identified 572 genes involved in phenotypic perturbations during mitosis in the human HeLa cell line (see Materials and Methods). The authors have used RNA interference to silence each of the ≈21000 human protein-coding genes. Based on high-throughput time-lapse microscopy, they were able to study detailed phenotypes and the dynamics of these perturbations at the cell population level. Phenotypical relevance is defined here as phenotypic deviations detected when compared to control cells. The set of 572 phenotypically relevant genes identified by the authors will henceforth be defined in this paper as mitotic hit (MH) genes and all other human protein coding genes will be defined as non-MH genes.

We have constructed and analyzed a molecular interaction network focused on biological processes and pathways related to mitosis and cell division. Our results demonstrate a relationship between the network location of MH genes and their phenotypic outcomes. Furthermore, we performed a pairwise, comparative analysis for conservation of human genes, interactions, as well as network motifs compared to a panel of 9 metazoan species. In which, MH genes and the interactions of their corresponding proteins were found to be significantly more conserved when compared to other genes. Fragmentation analysis of the network of conserved interactions showed that the MH interactions are important for the topological integrity of this conserved network. We report here, that MH genes participate exclusively in a specific class of evolutionarily conserved bistable network motifs, which are likely to contribute to network stability. This finding suggests that these genes are important for the functioning of cell division-related processes and therefore contribute to the phenotypic robustness of mitosis.

## Results

### Relationship between distinct types of phenotypic cell cycle perturbations and biological processes

Our analysis involved a biological network focusing on the phenotypic and regulatory aspects of cell division. We used the Kyoto Encyclopedia of Genes and Genomes (KEGG) as a resource to construct a molecular interaction network (MIN) consisting of functional and physical protein interactions (see Materials and Methods). Gene Ontology (GO) [19] enrichment analysis for genes in the KEGG MIN network revealed an enrichment of biological topics related to mitosis and cell division (Supporting information S1). For this analysis we used the complete set of human genes involved in protein interactions in KEGG as background. The MH genes were found to be distributed heterogeneously among KEGG MIN, although certain pathways and pathway classes are significantly enriched with these genes (Supporting information S2). This quality assessment of the network suggests that our KEGG MIN represents a suitable network for investigating the genotype-phenotype relationships of MH genes.

We investigated the relationship between the location of the MH genes in the KEGG MIN and the temporal phenotypic changes caused by the silencing of these genes. We performed a statistical analysis comparing the phenotypic distances between MH genes and their proximity to each other within the KEGG MIN. We grouped the phenotypic distances between MH genes on the basis of their corresponding network distances. For a given network distance *d*, group I consists of pairs having their network distances lower or equal to *d*, while group II consists of the remaining pairs (see Materials and Methods). We compared the difference between the phenotypic distances of pairs in I and II. A one-sided Wilcoxon rank-sum test was used to determine if the MH genes grouped by the shorter network distances (group I) are closer with respect to their phenotypic distances than the other MH genes (group II).

**Figure 1:**
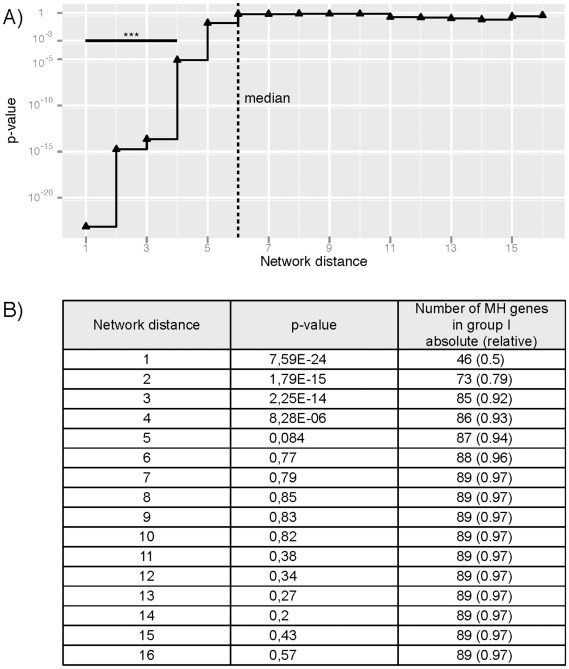
Relation between network and phenotypic distances in the KEGG MIN. Network and phenotypic distances for all pairs of MH genes in KEGG MIN were calculated (see Materials and Methods). The range of values for MH network distances is depicted on x-axis (Network distance). For each element *d* of this range the set of phenotypic distances was split into two parts: group I consists of phenotypic distances with corresponding network distance lower or equal to *d*; and group II consists of phenotypic distances with corresponding network distance greater than *d*. A) One-sided Wilcoxon rank-sum test was performed to determine whether the values of phenotypic distances in the first group are lower than in the second group. P-values are shown on the y-axis (p-value). The range of statistically significant values (***: p-value<0.001) is depicted by a horizontal bar. The dotted vertical bar indicates the median of observed network distances for MH genes. B) Table containing p-values displayed above and the number of MH genes in group I, absolute, and relative to the total number of genes in group I and II, respectively, for all analyzed values of network distance.

As shown in Figure 1A, the statistical significance, for differences between the two groups of phenotypic distances, decreases for increasing network distances. MH genes with a network distance lower or equal to four have a significantly closer phenotypic distance compared to the more distant MH genes. This result indicates a significant relationship between the network proximity of MH genes to each other and their phenotypic similarity. Figure 1B provides detailed information on the p-values of statistical tests for subsequent values of network distance. Also, the number of MH genes present in group I are given. These results show that for the majority of MH genes (86 of 92) the proximity of MH genes to each other in the network corresponds to a similarity in the phenotypic outcome.

**Figure 2:**
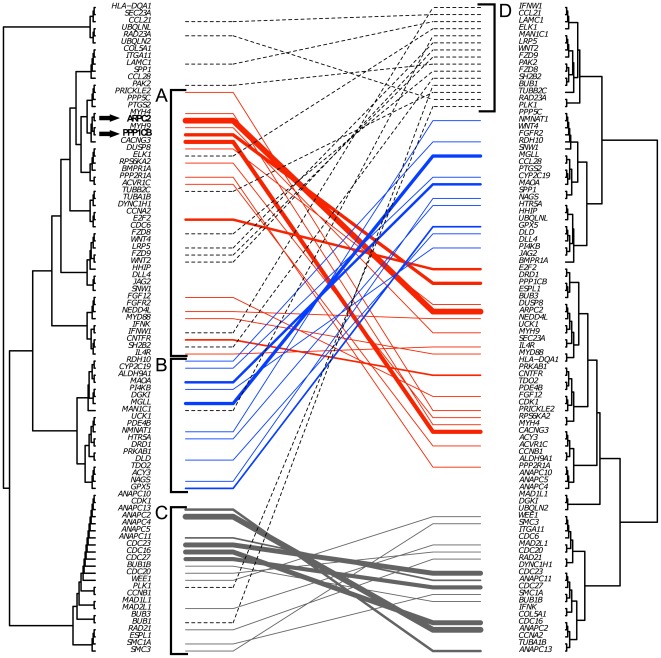
Mirrored trees. Comparison of similarity of mitotic hit genes regarding their location in KEGG MIN and the phenoprints observed after gene silencing. Left side tree represents shortest path between MH genes in the KEGG MIN; right side tree represents similarity measure for phenoprints of MH genes based on Hausdorff distance. A line connects a gene in a pair of corresponding clusters of both trees. Thickness of the line reflects the depth in the tree, on which considered gene remains within corresponding clusters on both sides.

To obtain more detailed information about the relationship between network topology and phenotype, we clustered the MH genes and illustrated the outcome using two tree dendrograms. One of the trees shows the distance between the MH genes in the KEGG MIN, whereas the other tree depicts the similarity of their phenoprints. In Figure 2, one can observe groups of genes that, on the one hand, are related by their phenoprints and on the other hand are located nearby in the KEGG MIN. Four clusters of genes can be visually distinguished. Cluster A corresponds to the MH genes implicated in signal transduction and cell junction (including ARPC2 and PPP1CB), which display a tight connection on both a network and phenotypic level. Cluster B corresponds to metabolism and cluster C corresponds to mitosis. Additionally, our analysis revealed a specific group of genes which are represented by cluster D. These genes are not mapped to a specific network area. They show a broad distribution over the whole KEGG MIN, and yet have similar phenotypic outcomes. The content of cluster D was found to be associated with cell surface receptors, receptor ligands and ECM molecules involved in “Notch signaling", “Wnt signaling" as well as “ECM-receptor interaction" pathways. Such genes represent the entry points to various intracellular signal transduction cascades and pathways but they are not located in close proximity to each other in the network. Our analysis revealed that these ligands and receptors display similar phenotypic characteristics and this in turn suggests downstream convergence of signaling events triggered by these receptors and ligands by implicating cross-talk at the level of signal transduction pathways. MH genes grouped in specific clusters display a relationship at the functional level, namely mitosis, metabolism, as well as signal transduction and cell junction.

We performed a statistical analysis to delineate the correspondence between network and phenotypic distances and validated this correspondence using the mirrored trees. Effectively, our analysis allowed to pinpoint a relationship between specific network regions representing biological processes and the phenotypic outcomes of MH genes in these regions.

### Evolutionary conservation of mitotic hit genes and their interactions

The following analysis step was undertaken to compare evolutionary conservation of MH and non-MH protein-coding genes, as well as their corresponding interactions (Table 1). We focused on the conservation of genes and compared their interactions between human and a panel of metazoan species. We applied an inter-species conservation cut-off of 70% for detecting evolutionary conserved genes as well as protein and gene regulatory interactions (see Materials and Methods). We define here the MH interactions, as human interactions that involved at least one MH gene. Correspondingly, non-MH interactions are human interactions that involve no MH gene.

**Table 1:** Evolutionary conservation of MH genes and interactions.

		HomoloGene		KEGG		Reactome	
	MH	Non-MH	MH	Non-MH	MH	Non-MH	
**All**	Conserved	398	11170	778	9952	2128	10614
	Non-conserved	165	6533	1261	21164	6754	53900
	p-value	0.0001		6.93e-09		4.54e-64	
**KEGG MIN**	Conserved	398	1804	675	8654	745	2057
	Non-conserved	165	592	1124	16418	2252	7070
	p-value	0.989		0.0054		0.005	
**Regulatory KEGG MIN**	Conserved	398	915	480	2970	519	1284
	Non-conserved	165	378	796	7129	1824	5469
	p-value	0.536		2.06e-09		0.0006	

Human genes and interactions are classified as evolutionary conserved or non-conserved using inter-species conservation cutoff of 70% (see Materials and Methods) for human protein-coding genes (HomoloGene), protein and gene regulatory interactions (KEGG and Reactome). The p-values are based on Fisher exact test for enrichment of MH compared to non-MH genes or interactions. Enrichment is tested for all available protein-coding genes and/or protein interactions (All); for cell division and mitosis-focused genes and/or interactions from KEGG MIN (KEGG MIN); and for the regulatory genes and/or interactions from KEGG MIN (Regulatory KEGG MIN).

Initially, we focused on comparing evolutionary conservation for the MH and non-MH genes and their respective protein and gene regulatory interactions. We used different data resources for assessing evolutionary conservation of protein coding genes and interactions, i.e. HomoloGene, KEGG and Reactome.

We aimed to delineate the evolutionary conservation of MH genes and their interactions by using all the protein coding genes and their respective protein and gene regulatory interactions (as described in the different data resources listed above.). Statistical analysis, based on the Fisher exact test, indicated a significant enrichment of MH genes (p-value = 0.0001) among conserved human protein-coding genes. Further analysis compared evolutionary conservation patterns at the level of MH and non-MH protein and gene regulatory interactions, which was based on interactions extracted from the KEGG database. A statistically significant conservation of MH interactions was found when compared to non-MH interactions (p-value = 6.93e-9). A similar analysis based on the Reactome protein interaction database allowed us to further validate this finding (p-value = 4.54e-64).

Subsequently, we wanted to clarify, whether the conservation patterns of MH genes and their interactions were conditioned only by their phenotypic relevance or in addition by their implication in mitosis-related processes. Two lists of genes and their interactions named KEGG MIN and the Regulatory KEGG MIN were constructed focusing on the biological processes and pathways relevant for cell division and mitosis (see Materials and Methods). We directly compared the MH and non-MH genes and their interactions from these two lists to see if the evolutionary conservation pattern observed for the MH genes and their interactions is distinctive compared to other mitosis-related genes and interactions.

Our results demonstrate no enrichment of conserved genes for the group of MH genes compared to non-MH genes (p-value = 0.989 and 0.536 for KEGG MIN and Regulatory KEGG MIN, respectively). In contrast, the MH interactions in comparison to other cell division-related interactions (non-MH interactions) from the KEGG MIN show a higher degree of conservation in metazoans (p-value = 0.0054 and 0.005 for KEGG and Reactome, respectively). Similarly, MH interactions in Regulatory KEGG MIN show a higher level of conservation across metazoans (p-value = 2.06e-9 and 0.0006 for KEGG and Reactome, respectively) when compared to other interactions.

We further validated these findings by applying other inter-species conservation cutoffs for human genes and protein interactions (60% and 80%, see Supporting information S3).

Taken together, our findings reveal a distinctive conservation pattern on a whole-genome scale for MH genes and their interactions compared to other protein-coding genes and their interactions. By focusing on cell division and mitosis related networks (KEGG MIN and Regulatory KEGG MIN), we show that MH interactions are significantly more conserved than non-MH interactions. In the same context, the evolutionary conserved genes are not enriched with MH genes compared to other protein coding genes. Therefore, our results suggest that evolutionary conserved MH interactions play a key functional role for the cellular phenotype during mitosis.

### Topological relevance of mitotic hit interactions in the conserved molecular interaction network

Statistical analysis suggests an enrichment of conserved interactions in the group of MH genes but it demonstrates nothing of their role in the corresponding network. An evolutionary conserved network helps to delineate a framework of collaborating proteins and interactions of functional relevance for mitosis. In this context, we evaluated the topological importance of conserved MH genes and their interactions within the evolutionary conserved KEGG MIN and Regulatory KEGG MIN by performing fragmentation analysis (see Materials and Methods). Fragmentation analysis allows assessment of the robustness of the whole network against targeted perturbations, i.e. removal of the MH genes or their interactions.

We based our analysis on the evolutionary conserved networks, namely KEGG MIN and Regulatory KEGG MIN. Distinct fragmentation analyses for nodes (MH genes) and edges (MH interactions) were performed (Table 2). The size of the giant component obtained by removing either the MH genes or their interactions, corresponds to 1383 nodes (KEGG MIN) and 628 nodes (Regulatory KEGG MIN). To assess the statistical relevance of these values, we performed a random network fragmentation based on the genes and their interactions, separately, for both networks. A one-sided Z-test revealed a significant decrease in the giant component size obtained by removal of MH interactions, when compared to random interaction removal, for both the KEGG MIN (p-value = 8.72e-10) as well as the Regulatory KEGG MIN (p-value = 6.83e-7). Interestingly, we didn't find a significant difference for MH genes when compared to random gene removal for either the KEGG MIN (p-value = 0.35) or the Regulatory KEGG MIN (p-value = 0.71).

**Table 2:** Fragmentation analysis of evolutionary conserved networks.

	Number of MH genes/interactions	Initial network size	GCS^1^ after removal of MH genes/interactions	GCS^1^ after random removal of genes/interactions: mean (s.d.^2^)	p-value^3^
KEGG MIN genes	64	1617	1383	1388.3 (13.95)	0.35
KEGG MIN interactions	544	1617	1383	1445.52 (10.3)	8.72e-10
Regulatory KEGG MIN genes	44	764	628	623.1 (8.8)	0.71
Regulatory KEGG MIN interactions	281	764	628	652.75 (5.12)	6.83e-7

GCS^1^: giant component size.

s.d.^2^: standard deviation.

p-value^3^: p-value based on the Z-score of the GCS after MH removal.

These results demonstrate that removal of MH interactions has a significantly higher impact on the network structure compared to random non-MH interactions (this observation holds for both the KEGG MIN and Regulatory KEGG MIN). However, they also show that the impact on the network topology, by removal of MH genes, is not significantly affected when compared to random gene removal for both evolutionary conserved networks. These results are in perfect concordance with the conservation patterns for MH genes and their interactions described in the previous section. By this analysis we show that evolutionary conserved MH interactions are important for the topological integrity of the conserved cell division and mitosis networks.

### Evolutionary conservation of network motifs implicating mitotic hit genes

**Figure 3:**
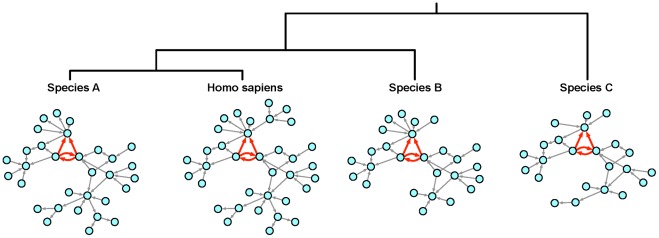
Concept of evolutionary motif conservation. Phylogenetic tree for different species and corresponding species-specific networks of conserved gene regulatory interactions compared to Homo sapiens. A network motif shared between regulatory networks in human and other species is highlighted in red.

Subsequently, we analyzed the implication of MH genes in evolutionary conserved network motifs. Figure 3 demonstrates the concept of our approach illustrating a network motif conserved within the human regulatory network and the corresponding networks of other species. Such a network motif might reflect a conserved functional characteristic emerging from the regulatory interactions among the implicated genes.

Considering the biological relevance of bistable motifs [20], we focused our analysis on this particular class of motifs in the Regulatory KEGG MIN. A bistable motif is defined as a regulatory network motif that can reside stably in either of its two steady states [20], [21]. We identified 6 distinct classes of bistable motifs of size 3, as well as 22 distinct classes of bistable motifs of size 4. These motif instances were analyzed for evolutionary conservation. We defined a motif instance as conserved if all of its interactions were conserved in the same set of at least seven species. Furthermore, a bistable MH motif instance is defined as encompassing at least one MH gene. Other motif instances are referred to as non-MH motif instances. The numbers of evolutionary conserved bistable motif instances in the groups of MH genes and non-MH genes are shown in Table 3 along with the number of involved genes and their protein interactions.

**Table 3:** Evolutionary conserved bistable motifs.

		Bistable motifs of size 3	Bistable motifs of size 4
**MH**	Number of motif instances	292	1213
	Number of interactions	144	156
	Number of genes	19	28
**Non-MH**	Number of motif instances	3	2
	Number of interactions	9	8
	Number of genes	5	5
p-value (Fisher exact test)^1^		1.15e-11	

1 p-value is based on Fisher exact test for enrichment of MH genes within bistable motifs.

It is striking that evolutionary conserved bistable motifs of size 3 and 4 are significantly enriched for MH genes (p-value = 1.15e-11) when compared to non-MH genes. Moreover, we found 292 and 1213 bistable MH motif instances, in contrast to 3 and 2 non-MH motif instances for network motifs of size 3 and 4, respectively. This finding indicates the biological relevance of evolutionary conserved bistable network motifs for cell cycle progression.

### Topological and functional characteristics of evolutionary conserved bistable motifs

**Figure 4:**
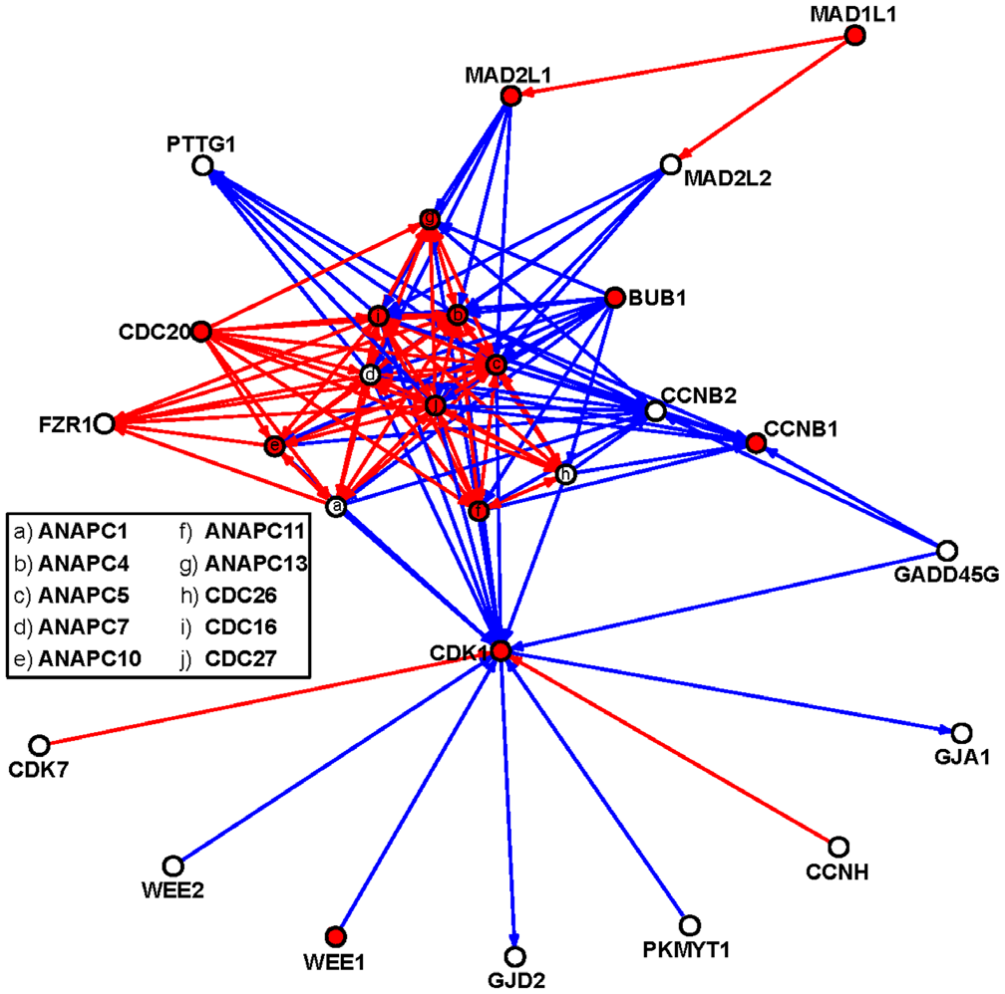
Aggregation of evolutionary conserved bistable motifs. Aggregation of instances of bistable motifs forms a cluster in the evolutionary conserved KEGG molecular interaction network. The node labels correspond to the official gene symbols. MH genes are depicted as red nodes. Activation and inhibition edges are depicted in red and blue, respectively.

As a next step, we explored the spatial distribution of conserved bistable network motifs in the Regulatory KEGG MIN. The bistable motifs of size 3 and 4 form a cluster within the network (see Figure 4). To provide statistical support to this statement we have compared local clustering coefficients [22] of genes from the cluster to the rest of the genes in the Regulatory KEGG MIN using Wilcoxon rank-sum test. We found that the genes within the cluster display significantly higher (p-value = 1.11e-15) local clustering coefficients.

This cluster encompasses 28 genes including 14 MH genes implicated in 1505 instances of MH motifs and 5 instances of non-MH motifs (Supporting information S1). No genes or regulatory interactions in the motif cluster are exclusively associated to non-MH motif instances and this suggests a tight coupling of their functional properties with MH motifs in the bistable network motif agglomerate. Interestingly, the bistable MH motifs in this cluster represent exclusively motifs encompassing a positive feedback loop (Supporting information S3). This characteristic delineates the biological relevance of positive feedback loops for mitosis.

To investigate the biological coherence of MH motif instances implicated in the bistable motif cluster, we performed a functional enrichment analysis by comparing genes implicated in bistable MH motif instances to all genes in the Regulatory KEGG MIN. By considering GO annotations, it emerged that the genes from the motif cluster represent a functionally homogenous group (Supporting information S1). The top ranked GO terms, ordered by LOD score, include “mitotic anaphase", “anaphase", “anaphase-promoting complex-dependent proteasomal ubiquitin-dependent protein catabolic process", “nuclear ubiquitin ligase complex" and “anaphase-promoting complex" among others. This result indicates a tight functional association of the bistable MH motif cluster with the anaphase-promoting complex/cyclosome (APC/C).

Finally, we characterized the importance of evolutionary conserved bistable network motifs in pathway cross-talk (see Supporting information S3). Our results highlight the implication of selected cell communication (Gap junction, hsa04540) and signaling pathways (MAPK (hsa04010) and p53 (hsa04115)) involved in cross-talk with the “cell cycle" pathway (hsa04110).

## Discussion

The main goal of this study was to develop an understanding of the link between gene silencing and the resulting cellular phenotypes, in the context of cell division. It should be emphasized that our aim in the context of the genotype-phenotype relationship was to provide characterization, at the network level, of the phenotypically relevant mitosis genes in humans. We based our analysis on a genome-wide experimental study, which identified 572 genes affecting phenotypic outcomes of HeLa cells during mitosis [18]. In order to identify the common biological and network topology characteristics of these phenotypically relevant genes, we combined a molecular interaction network analysis with evolutionary information.

A molecular interaction network related to cell division and mitosis was created in order to analyze resulting phenotypic perturbations in this biological context. Our initial results revealed a significant enrichment of biological topics related to mitosis and cell division in this KEGG-based functional and physical protein interaction network. By focusing on pathway annotation we were able to show that MH genes are implicated in various pathways and pathway classes and are not exclusively related to the cell cycle pathway. Interestingly, this analysis revealed the importance of metabolic pathways for phenotypic perturbations of mitosis. Thus, the selected list of KEGG pathways reflects a relevant group of pathways and corresponding protein interactions, which are well suited for analyzing the functional and topological characteristics of MH genes at the network level.

Subsequent analysis revealed a relationship between the proximity of MH genes at the level of network representing specific cellular processes, and the cell population-wide phenotypic changes over time caused by gene silencing. This suggests that perturbations in specific network areas may result in similar phenotypic outcomes. One possible explanation for this observation is the lack of efficient molecular backup mechanisms that compensate for the effects of gene knock-down.

In concordance with our findings, other research has shown that orthologous phenotypes allow identification of evolutionary conserved subnetworks implicating genes relevant for a specific phenotype in humans [23]. As an example, the actin related protein (Arp) 2/3 complex member ARPC2 and the protein phosphatase 1 catalytic subunit PPP1CB show a close location in the molecular interaction network as well as a similar phenotypic outcome following gene knock-down (see Figure 2). The involvement of Arp2/3 complex was reported to cause the delay in mitosis progression and increase in polyploidy [24], [25]. Protein phosphatase 1 was observed in conjunction with increased polyploidy and delayed mitosis in Long Evans Cinnamon rat, an animal model for human Wilson's disease [26]. Our results suggest that PPP1CB might act as a trigger for the observed mitotic delay and polyploidy in this model and by considering the proximity with ARPC2 on a phenotypic and network level, we propose the existence of common molecular mechanisms for mediating these phenotypic effects.

We wanted our conclusions to be independent from specific characteristics of the HeLa cell line. Therefore, we have based our analysis on evolutionary conservation by focusing on individual genes, their interactions, network motifs as well as pathway cross-talk. As a consequence we were able to pinpoint a tight correlation between evolutionary conservation of the MH genes, as well as the protein interactions, and perturbation of mitosis at the phenotypic level. It is well known that proteins of the cell cycle machinery are functionally and structurally conserved across eukaryotes, even though their regulation differs between organisms [27], [28]. Interestingly, our results revealed that protein and gene regulatory interactions of phenotypically relevant genes show a higher degree of conservation when compared to other cell division related interactions. In fact, the pattern of evolutionary conservation of protein and gene regulatory interactions is a distinctive characteristic of the phenotypically relevant genes for mitosis. This suggests the importance of these interactions for the phenotypic outcome of the cell during mitosis. To further examine the properties of these conserved protein interactions,we used them to construct an evolutionary conserved network. Following network fragmentation analysis for this conserved network, we found that the conserved MH interactions are important for the topological network integrity, thus suggesting their functional relevance.

Network motifs represent the functional building blocks of regulatory networks [29]. Their usage under different conditions, (including the cell cycle among others), has already been investigated [30]. It has previously been proposed that the biological relevance of network motifs [31] is demonstrated by the high level of evolutionary conservation of network motif constituents in the yeast protein interactome [14], as well as the evolutionary convergence towards the same types of motifs in transcriptional regulatory networks across different species [32], [33]. In this context, it has been suggested that the convergent evolution of network motifs contributes to the optimization of evolutionary fitness by adjusting gene expression levels [34].

It has been shown that the robustness of network motifs to perturbations is a relevant determinant for the structure of biological networks [35]. Furthermore, it has been proposed that bistable motifs play a key role in the stabilization of normal and diseased cellular states, as well as the transition among them [20]. Here we extend this concept by analyzing the role of evolutionary conserved bistable motifs (henceforth called bistable motifs) in deregulating mitosis-related processes. We suggest that specific perturbations, i.e. silencing of genes that are implicated in bistable motifs in the cell division network, may have detrimental effects for the phenotypic stability of mitosis. Following this rationale, we identified the enrichment of relevant genes for phenotypic perturbations of mitosis in bistable motifs. Moreover, we were able to determine that nearly all of the bistable motifs implicate at least one MH gene. Our study shows that evolutionary conserved bistable motifs and the implicated genes are highly relevant for determining mitosis-related phenotypic outcomes. Interestingly, evolutionary conserved bistable MH motif instances in our network were found to agglomerate in a network motif cluster. These findings are in concordance with a previous study that described the grouping of motif instances into network clusters with specific biological functions [31]. Indeed, by performing functional enrichment analysis for the group of genes implicated in bistable MH motif instances in our network motif cluster, we were able to reveal a tight biological connection to anaphase and more precisely anaphase-promoting complex-dependent protein catabolism.

Additionally, the bistable MH motifs detected in our study exclusively implicate positive feedback loops. The importance of positive feedback loops in the control of different cell cycle phases in *Saccharomyces cerevisiae* has previously been described [36], [37]. These types of motifs have been suggested to be a key factor in keeping the cell cycle machinery working in a synchronized manner [38]. Moreover, a recent study has proposed a model, in which positive feedback circuits, by acting as one-way toggle switches, allow the metaphase-anaphase switch to become irreversible [39]. Another important aspect of positive feedback loops, when coupled with negative feedback loops, relates to the tuning of the frequency of the biological response by maintaining a constant amplitude in the cell cycle [40]. Thus, our findings stress the importance of bistable motifs, and in particular positive feedback loops, as key structures for maintaining the phenotypic integrity of the cell during mitosis.

Many of the MH genes implicated in conserved bistable motifs were described as key regulators for cell cycle progression and more specifically mitosis. Among them we find cyclin B1 and its kinase partner CDK1, the main regulators of mitotic entry [41]. A recent review by Moseley and Nurse gives an excellent survey on bidirectional CDK1-cellular morphology related signaling in the model organism yeast, suggesting the crucial role of CDK1 and its interactions at the morphological level of the cell cycle [42]. Considering the functional enrichment of APC/C related processes and their functions in our network motif cluster, it might be important to highlight the biological relevance of the APC/C, as having a key role in mediating the exit from mitosis, by regulating the degradation of mitotic cyclins [43] and other key mitotic factors [44].

Interestingly, we observed several bistable MH motifs implicated in pathway cross-talk. This cross-talk involves, on the one hand, the cell cycle pathway and on the other p53 signaling, mitogen-activated protein kinase (MAPK) as well as gap junction pathways. The biological relevance of bidirectional pathway cross-talk linking connexins and related gap junction communication pathways to the regulation of cell proliferation was described [45]. It is well known that the MAPK pathway is one of the most crucial signal transduction pathways implicated in cell proliferation, cell differentiation and cell cycle regulation [46]. The mechanisms and biological impact of pathway cross-talk between MAPK and cell cycle pathways have been thoroughly studied [47]–[50]. The p53 tumor suppressor pathway is activated in response to various intrinsic and extrinsic stress signals [51], [52] leading to cell cycle arrest, cellular senescence or apoptosis [53]–[55]. The specific implication of network motifs, namely feedback loops, in the p53 signaling pathway cross-talk in the cell cycle was recently reviewed [56]. These observations in conjunction with our findings strongly support the critical role for bistable MH motifs in pathway cross-talk and their effects on the cell cycle.

Whether these findings represent a general footprint for phenotypic perturbations of key cellular processes or a specific pattern for mitosis in the metazoan species used in this study, remains to be determined in the future. Even though our approach allowed us to provide relevant biological information, it has shortcomings. The availability, completeness and quality of protein interaction datasets are limited [57]. Currently there is a lack of large-scale interactome and gene regulatory network datasets allowing accurate cross-species comparison for evolutionary conservation. It is important to stress that orthologous protein interactions provided by KEGG and Reactome are implicitly inferred based on the evolutionary conservation of genes that interact at the protein level [58], [59]. Hence, our study was limited to the evolutionary retention of genes, as well as interacting protein pairs, when focusing on evolutionary conservation of protein interactions and network motifs. The improvement of completeness and quality of species-wide protein interaction maps and gene regulatory network data might help to obtain further insights, especially in the context of motifs and their relevance for network stability, in conjunction with the outcome at the level of cellular phenotype. To further strengthen our findings, the precise role of the MH genes (in the context of motif dynamics) as well as their relevance for network stability, needs to be elucidated in more depth. This may provide further insights into the link between genotype and phenotype.

In summary, molecular interaction network analysis along with evolutionary information allowed us to characterize key genes for mitosis whose silencing significantly affects this process at a phenotypic level. Our analysis revealed network topology characteristics and the distinctive evolutionary conservation pattern of protein interactions. We have highlighted the key role of network motifs in the context of evolution for providing phenotypic robustness to mitosis. This study extends the knowledge base of genotype-phenotype relationships for cell division in humans.

## Materials and Methods

### Conversion of gene identifiers from different datasets

To facilitate data integration from different sources, gene identifiers (IDs) were converted if necessary to National Center for Biotechnology Information (NCBI) Entrez Gene IDs. Concerning the MH gene dataset [18], Ensembl Gene IDs for the set of 572 MH genes were obtained from the Mitocheck website (http://www.mitocheck.org) on July 22 2010. Ensembl IDs were converted to NCBI Entrez Gene IDs using the Bioconductor [60] package biomaRt (version 2.8.0) which accesses online the Ensembl mart (release 61) web service. The Whitfield dataset containing genes that are periodically expressed during cell cycle in HeLa cells [61] was downloaded from the corresponding website (http://genome-www.stanford.edu/Human-CellCycle/HeLa/).We extracted the GenBank and RefSeq IDs for each of the 1134 periodically expressed clones and mapped these to Entrez Gene IDs using UniGene (build 226).

### Gene Ontology enrichment analysis

GO based functional enrichment analysis for groups of genes was performed with FuncAssociate 2.0 software [62] using default parameter settings. The set of genes used as background is indicated in the corresponding Results section.

### Protein-protein interaction and gene regulatory networks

MH genes are involved in mitotic perturbations in human HeLa cancer cell line. Our main goal was to provide general conclusions on evolutionary conservation and network topology characteristics of MH genes in human. Based on our expert knowledge, we selected a list of KEGG pathways that reflect phenotypic as well as regulatory aspects of mitosis and cell division in human. Therefore, the following filter was applied: the resulting list of pathways should reflect metabolic aspects, genetic and environmental processing, cellular processes and physiological systems impacting mitosis and cell division independent of pathology, cell lineage and organ specific characteristics. To further strengthen the focus on mitosis and cell division, we restricted the resulting pathways to those involving at least one gene identified as periodically expressed in the cell cycle [61] (Supporting information S1).

The corresponding KEGG pathways in human [59], [63] were obtained on January 21st 2011 by FTP. The protein interactions, including protein-protein interactions, gene expression and enzyme relations were extracted using Bioconductor [60] package KEGGSOAP. The resulting KEGG MIN contains in total 2539 proteins and 22450 interactions, including 92 MH genes (Supporting information S1).

Additionally, the gene regulatory part of this network (Regulatory KEGG MIN) was obtained by filtering non-relevant interactions. Hence, only the following types of interactions were retained: activation, expression and positive indirect effect define the set of positive regulatory interactions while inhibition, repression and negative indirect effect define the group of negative regulatory interactions. The Regulatory KEGG MIN (Supporting information S1) contains in total 1388 nodes, including 63 MH genes, connected by 6508 directed regulatory interactions.

Furthermore, we derived the evolutionary conserved KEGG MIN and Regulatory KEGG MIN by focusing on evolutionary conserved human interactions in the KEGG MIN and Regulatory KEGG MIN, respectively (see Materials and Methods, section “Evolutionary conserved human protein interactions"). An inter-species conservation cutoff of 70% was applied. The conserved KEGG MIN encompasses a total of 1617 proteins and 8619 edges, including 64 MH genes. The conserved Regulatory KEGG MIN encompasses a total of 764 proteins and 2372 edges, including 44 MH genes.

### Enrichment analysis for pathway and pathway class participation

The implication of genes in pathways was extracted from KEGG pathways using the KEGGSOAP package. Pathway classification (Supporting information S1) was retrieved from the KEGG website (http://www.kegg.jp/). Similarly, directed links between pathways were retrieved in order to construct the KEGG MIN-based network of pathways and pathway classes (Supporting information S1).

Enrichment analysis for assessing the strength of association between MH genes and pathways respectively pathway classes in the KEGG MIN was performed using Fisher exact test. The list of all genes in the KEGG MIN was used as background.

### Relationship between network and phenotypic distances

The phenotypic distance between MH genes was calculated based on the seven phenotypic outcome classes, which reflect phenotypic outcome following perturbation by gene silencing [18]. These phenotypic classes are as follows: “Mitotic delay", “Binuclear", “Polylobed", “Grape", “Large", “Dynamic" and “Cell death". The phenotypic changes in the cell population over time (phenoprints) are represented as trajectories in a 7-dimensional space [64]. The distance between trajectories was calculated using Hausdorff distance, an approach proposed to analyze multidimensional time-series data [65]. The network distance is calculated as the shortest path between the MH genes in the KEGG MIN.

Network and phenotypic distances for all pairs of MH genes were calculated as described above. A specific range of values was observed for the network distance. For each element *d* in this range the set of phenotypic distances was split into two parts. The first group (group I) consisted of phenotypic distances with corresponding network distance lower or equal to *d*. The second group (group II) consisted of phenotypic distances with corresponding network distance greater than *d*. One-sided Wilcoxon rank-sum test was performed to determine whether the median of phenotypic distances in group I is lower than in group II.

### Mirrored trees

The mirrored trees were constructed for the 92 MH genes present in KEGG MIN. The left tree corresponds to the shortest path between the MH genes in the KEGG MIN. The right tree corresponds to the distance between the phenoprints for MH genes based on Hausdorff distance, as described in the previous section. Results of both distance metrics were scaled and normalized to the [0,1] interval and clustered with Ward hierarchical clustering. The results were processed using the R package ape [66].

### Evolutionary conservation of human protein-coding genes

Based on the information contained in HomoloGene database (build 64) [67], evolutionary conservation, for the set of human protein-coding genes, was determined by focusing on a panel of nine metazoan species, including nematode (*Caenorhabditis elegans*), fruit fly (*Drosophila melanogaster*), zebrafish (*Danio rerio*), chicken (*Gallus gallus*), chimpanzee (*Pan troglodytes*), house mouse (*Mus musculus*), Norway rat (*Rattus norvegicus*), dog (*Canis lupus familiaris*) and cattle (*Bos taurus*). The corresponding phylogenetic tree is presented in Supporting information S2.

We define here a conserved gene as, a human gene that shares homologs in at least 70% of the considered species. Thus, by applying this inter-species conservation cutoff of 70%, the protein-coding genes were classified into two distinct groups: conserved and non-conserved. Evolutionary conservation of genes was assessed based on different sets of protein-coding genes extracted from HomoloGene: “All", “KEGG MIN" and “Regulatory KEGG MIN". “All" corresponds to protein-coding genes on a global biological level, which means that all protein-coding genes available in HomoloGene resource were considered for further analysis. “KEGG MIN" and “Regulatory KEGG MIN" correspond to lists of protein-coding genes in a cell-division or mitosis restricted context (see Material and Methods, section “Protein-protein interaction and gene regulatory networks"). Only the protein-coding genes from the HomoloGene database which implicate protein-coding genes found in the corresponding networks (KEGG MIN and Regulatory KEGG MIN) were considered for further analysis. The genes in the corresponding lists were grouped according to their phenotypical relevance (MH and non-MH genes). A Fisher exact test based on the resulting contingency tables was performed to compute the statistical significance of enrichment for MH genes in the group of conserved compared to non-conserved for the corresponding list of protein-coding genes (“All", “KEGG MIN" and “Regulatory KEGG MIN").

The evolutionary conservation of genes was additionally analyzed for inter-species conservation cutoffs of 60% and 80%. The results can be found in Supporting information S3.

### Evolutionary conserved human protein interactions

Similarly, as described in Materials and Methods, section “Protein-protein interaction and gene regulatory networks", we extracted functional and physical protein interaction data from the organism specific pathways for the group of nine species mentioned in Material and Methods, section “Homologous genes". The organism-specific pathways were downloaded from KEGG FTP server on January 21st 2011. In the context of protein interactions, a conserved human interaction is defined here by the evolutionary retention of the interacting protein pair in at least 70% of the considered species. This inter-species conservation cutoff of 70% allowed us to classify the protein interactions into two distinct groups: conserved and non-conserved. Furthermore, they were classified as MH and non-MH interactions. MH interactions are defined here as human interactions that implicate at least one MH gene. Non-MH interactions are defined here as human interactions that implicate no MH gene. Evolutionary conservation of interactions was assessed based on different sets of interactions extracted from KEGG: “All", “KEGG MIN" and “Regulatory KEGG MIN". “All" corresponds to interactions among protein-coding genes on a global biological level, meaning all protein interactions available in KEGG resource were considered for further analysis. “KEGG MIN" and “Regulatory KEGG MIN" correspond to lists of protein-coding genes in a cell-division/mitosis restricted context. Only the protein interactions from KEGG database which implicate the protein-coding genes found in the corresponding networks, i.e. KEGG MIN and Regulatory KEGG MIN (see Material and Methods, section “Protein-protein interaction and gene regulatory networks"), were considered for further analysis. The protein interactions in the resulting lists were classified as MH and non-MH interactions, according to the implication of phenotypically relevant genes. A Fisher exact test based on the resulting contingency tables was performed to compute the statistical significance of enrichment for MH interactions in the group of conserved compared to non-conserved for the respective list of interactions (“All", “KEGG MIN" and “Regulatory KEGG MIN").

As an additional data-source for evolutionary conserved human interactions, we downloaded from the Reactome website (http://www.reactome.org/) on February 2nd 2011 the data file containing the full listing of protein-protein interactions in Reactome [58], [68], including Homo sapiens and other species. Seven out of the nine previously selected species exist in Reactome: Bos taurus, Caenorhabditis elegans, Danio rerio, Drosophila melanogaster, Gallus gallus, Mus Musculus and Rattus norvegicus. As described in the previous paragraph for KEGG interaction data, orthologs for human interactions, were determined by comparing pairwise, the human interactome to the interactome of the seven species. Similarly as for KEGG (see previous paragraph), applying an inter-species conservation cutoff of 70% allowed classifying protein interactions into two distinct groups: conserved and non-conserved. Evolutionary conservation of interactions was assessed based on different sets of interactions extracted from Reactome: “All", “KEGG MIN" and “Regulatory KEGG MIN". “All" corresponds to interactions among protein-coding genes on a global biological level, meaning all protein interactions available in Reactome resource were considered for further analysis. “KEGG MIN" and “Regulatory KEGG MIN" correspond to lists of protein-coding genes in a cell-division/mitosis restricted context. Only the protein interactions from the Reactome database which implicate protein-coding genes found in the corresponding networks, i.e. KEGG MIN and Regulatory KEGG MIN (see Material and Methods, section “Protein-protein interaction and gene regulatory networks"), were considered for further analysis. The protein interactions in the resulting lists were classified as MH and non-MH interactions, according to the implication of phenotypically relevant genes. A Fisher exact test based on the resulting contingency tables was performed to compute the statistical significance of enrichment for MH interactions in the group of conserved compared to non-conserved for the respective list of interactions (“All", “KEGG MIN" and “Regulatory KEGG MIN").

The evolutionary conservation of human protein interactions in KEGG and Reactome was additionally analyzed for inter-species conservation cutoffs of 60% and 80%. The results can be found in Supporting information S3.

### Fragmentation analysis for conserved networks

Fragmentation analysis was performed according to the approach proposed by Dobrin and collaborators [31]. The networks used here, were the evolutionary conserved KEGG MIN and Regulatory KEGG MIN. Fragmentation analysis was performed separately for genes and interactions in both networks. For each network, the size of the resulting giant component was determined after removing the complete set of conserved MH genes or interactions.

To evaluate the impact of MH gene or interaction removal on the corresponding network, we performed random network fragmentation as follows: An equal amount of randomly chosen conserved genes or interactions were removed from the network. The size of the resulting giant component was determined. This process was repeated 5000 times. Mean and standard deviations were computed for the 5000 repeats. A one-sided Z-test was performed to determine if a significant decrease in giant component size was obtained after removing the complete set of MH genes or interactions compared to random.

### Conservation analysis for network motifs

Fanmod software [69] was used to search for motifs in the Regulatory KEGG MIN. The difference between activation and inhibition was achieved via separate edge colors for activation and inhibition-related edges. Motif detection was performed using the software default parameters. Motifs having more nodes than edges were not considered for further analysis to remove simplistic motifs. Default statistical cutoffs for detecting significant network motifs were used (Z-score> = 2, and p-value< = 0.05).

For each edge in the Regulatory KEGG MIN, the evolutionary conservation of human interactions was determined by applying an inter-species conservation cutoff of 70%, as described above for KEGG protein interactions. The motif instances where all edges were detected as conserved were considered as evolutionary conserved.

Genes in the Regulatory KEGG MIN were grouped according to their phenotypical relevance (MH and non-MH genes). Furthermore, these genes were classified on the basis of their participation in evolutionary conserved bistable motifs. A Fisher exact test based on the resulting contingency table was performed to compute the statistical significance of the association between MH genes and evolutionary conserved bistable network motifs.

## Supporting Information

Supporting Information S1 **Supporting tables with structure and annotation of analyzed networks.**
(XLS)

Supporting Information S2 **Supporting figures with additional details for distribution of MH genes within pathways and pathway classes, and phylogenetic tree for analyzed metazoan species.**
(PDF)

Supporting Information S3 **Supporting tables with complementary analysis of evolutionary conservation, conserved bistable motifs and pathway crosstalk in evolutionary conserved network.**
(PDF)
