# Supplementary material for: Evolutionary Conservation and Network Structure Characterize Genes of Phenotypic Relevance for Mitosis in Human
Source: PLoS One. 2012 May 2;7(5):e36488. doi: 10.1371/journal.pone.0036488 (PMC3342260; doi:10.1371/journal.pone.0036488)
Supplement: Supporting Information S2 — Supporting figures with additional details for distribution of MH genes within pathways and pathway classes, and phylogenetic tree for analyzed metazoan species. (PDF) [file pone.0036488.s002.pdf]

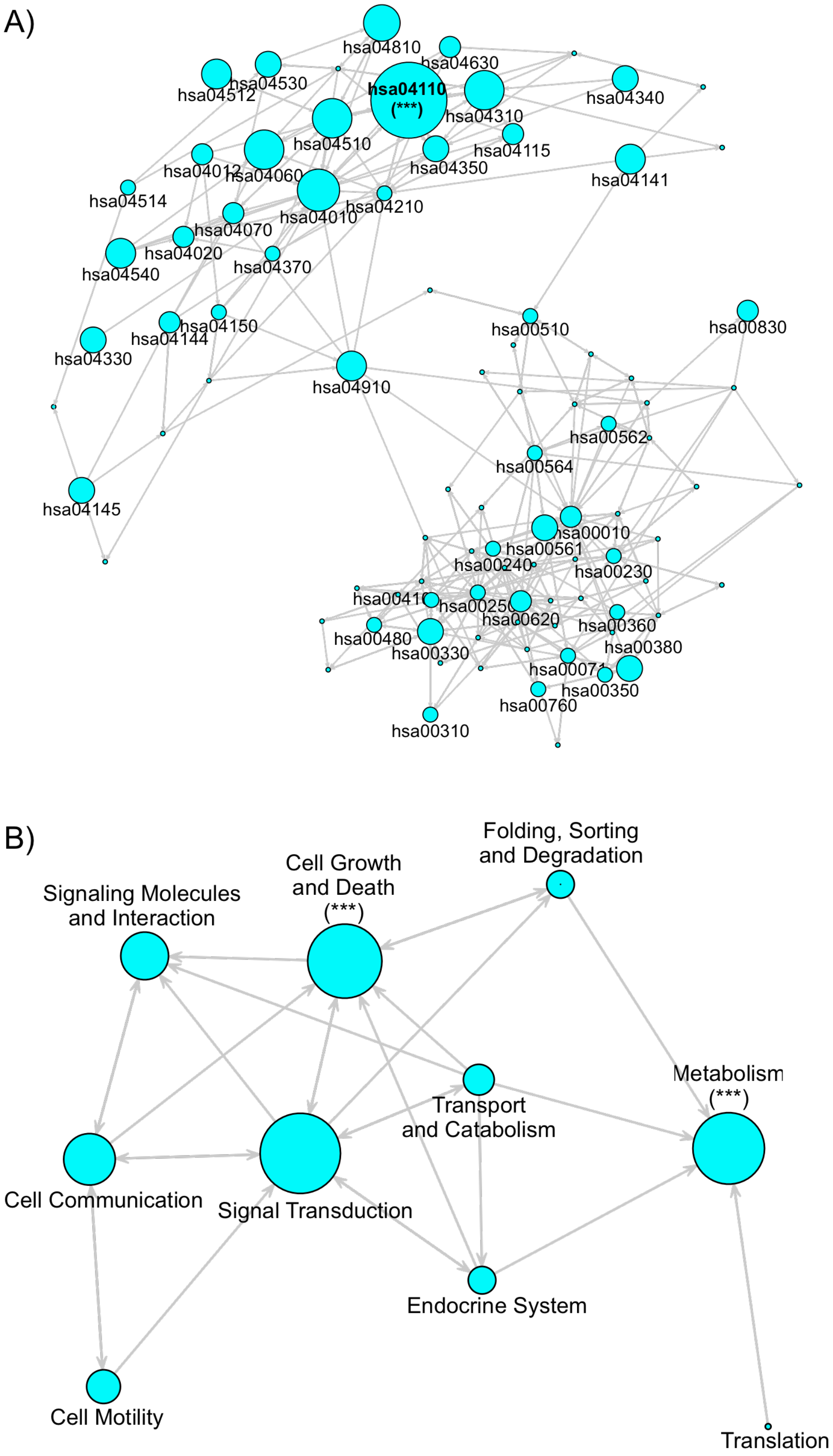

Figure S1: Distribution of mitotic hit genes across pathways and pathway classes. Distribution of MH genes across pathways, marked by their KEGG pathway ids (A) and KEGG pathway classes (B) in the KEGG molecular interaction network. Corresponding KEGG pathway or pathway class is indicated for each node. Node size is proportional to the number of MH genes within a pathway or a pathway class. Statistically significant enrichment of MH genes is shown (***: p-value<0.001).

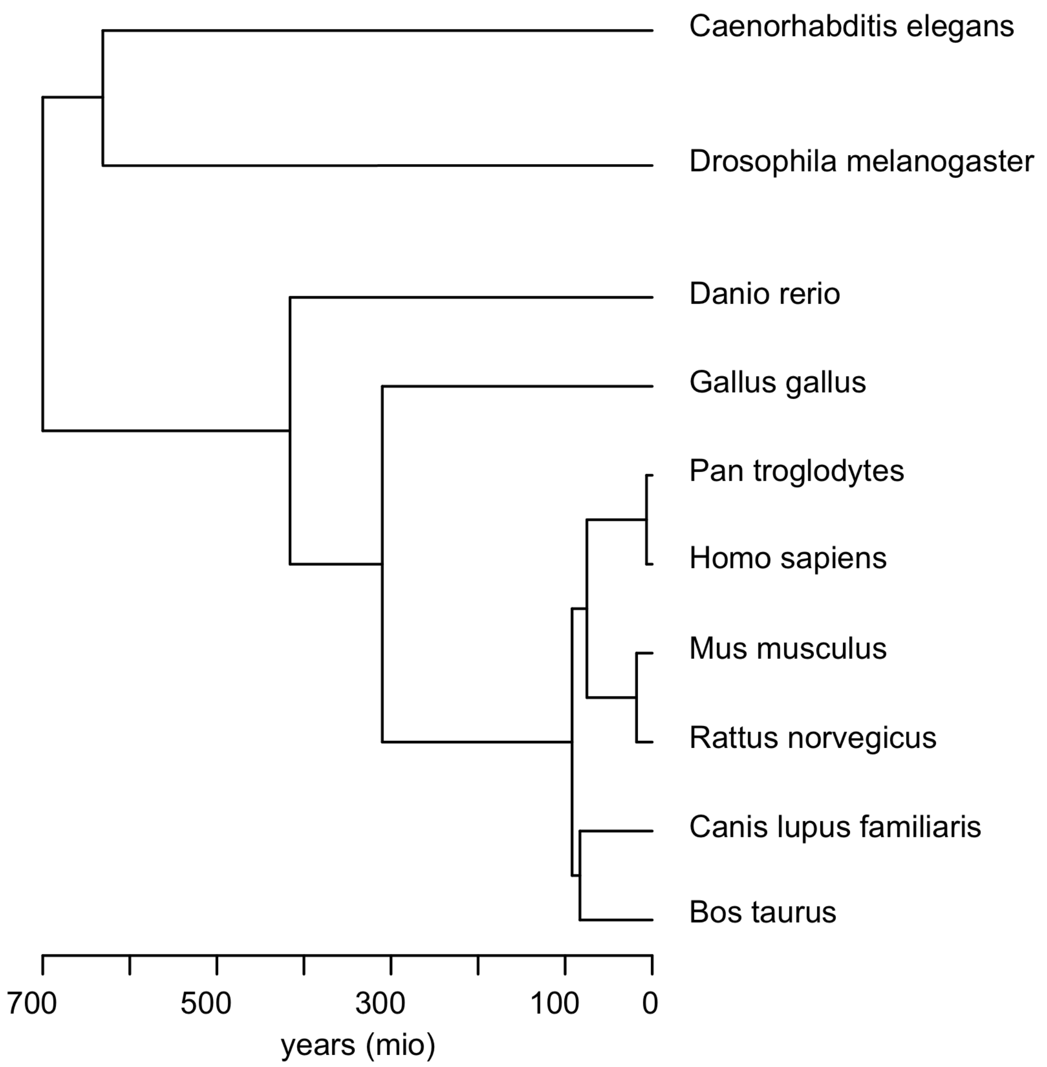

Figure S2: Phylogenetic tree. Phylogenetic relationship for the 10 metazoan species used in this study. Divergence time and branching order were taken from [1].

1. Ponting CP (2008) The functional repertoires of metazoan genomes. Nature reviews Genetics 9: 689–698.
