# Supplementary material for: Evolutionary Conservation and Network Structure Characterize Genes of Phenotypic Relevance for Mitosis in Human
Source: PLoS One. 2012 May 2;7(5):e36488. doi: 10.1371/journal.pone.0036488 (PMC3342260; doi:10.1371/journal.pone.0036488)
Supplement: Supporting Information S3 — Supporting tables with complementary analysis of evolutionary conservation, conserved bistable motifs and pathway crosstalk in evolutionary conserved network. (PDF) [file pone.0036488.s003.pdf]

Table S11: Evolutionary conservation of MH genes and interactions for 60% of species.

		Homologene		KEGG		Reactome	
		MH	Non-MH	MH	Non-MH	MH	Non-MH
All	Conserved	487	13879	1371	17887	4098	23730
	Non-conserved	76	3824	668	13229	4784	40784
	p-value	8.16e-07		1.26e-18		4.36e-64	
KEGG MIN	Conserved	487	2102	1205	15132	1462	4337
	Non-conserved	76	294	594	9940	1535	4790
	p-value	0.807		1.13e-08		0.119	
Regulatory KEGG MIN	Conserved	487	1115	896	5937	1091	3001
	Non-conserved	76	178	380	4162	1252	3752
	p-value	0.471		9.18e-16		0.0395	

MH – mitotic-hit
All – comparison of MH genes or interactions to all genes or interactions present in respective database
KEGG MIN – comparison of MH genes or interactions to genes or interactions present involving genes in cell cycle-related KEGG molecular interaction network

Table S12: Evolutionary conservation of MH genes and interactions for 80% of species.

		Homologene		KEGG		Reactome	
		MH	Non-MH	MH	Non-MH	MH	Non-MH
**All**	**Conserved**	271	7521	281	4054	785	3101
	**Non-conserved**	292	10191	1758	27062	8097	61413
	**p-value**	0.0041		0.173		1.26e-49	
**KEGG MIN**	**Conserved**	271	1264	230	3402	270	589
	**Non-conserved**	292	1132	1569	21670	2727	8538
	**p-value**	0.978		0.835		2.31e-06	
**Regulatory KEGG MIN**	**Conserved**	271	592	156	955	168	307
	**Non-conserved**	292	701	1120	9144	2175	6446
	**p-value**	0.189		0.00131		1.18e-06	

MH – mitotic-hit
All – comparison of MH genes or interactions to all genes or interactions present in respective database
KEGG MIN – comparison of MH genes or interactions to genes or interactions present involving genes in cell cycle-related KEGG molecular interaction network

Text S1: Functional characterization of evolutionary conserved bistable motifs

To gain insight into the functional characteristics of the bistable MH and non-MH motif instances in the network motif cluster, the clusters were classified into negative and positive feedback motifs. Negative feedback motifs involve at least one negative feedback loop, while positive feedback motifs incorporate exclusively positive feedback loops. The bistable MH motifs in the cluster represent exclusively motifs encompassing a positive feedback loop (292 instances for motifs of size 3 and 1213 motif instances for motifs of size 4, Supporting information S1). Bistable non-MH motifs encompass two instances of negative feedback loop motifs for motifs of both size 3 and 4 and one instance of positive feedback loop motif. These results indicate the importance of MH genes in evolutionary conserved bistable motifs. They also delineate the biological relevance of positive feedback loops for mitosis.

Text S2: Functional relevance of pathway cross-talk at the level of bistable motifs implicating mitotic hit genes

We wanted to characterize the importance of bistable network motifs in pathway cross-talk. We define here as cross-talk motif instance, a motif instance having genes associated with multiple pathways. A large number of MH instances are implicated in pathway cross-talk, linking pathways including p53 signaling (hsa04115), MAPK signaling pathway (hsa04010) and Gap Junction (hsa04540) to cell cycle (hsa04110), see Supporting information S1. For motifs of size 3, 95 out of 295 motif instances are implicated in cross-talk, while for motifs of size 4, 782 out of 1215 motif instances are mediating pathway cross-talk. These results point out the biological relevance of bistable MH motifs in signaling pathway cross-talk.
